# Smartphone-Based High-Throughput Fluorimetric Assay for Histidine Quantification in Human Urine Using 96-Well Plates

**DOI:** 10.3390/molecules28176205

**Published:** 2023-08-23

**Authors:** Dimitrios Baltzis, George Z. Tsogas, Constantinos K. Zacharis, Paraskevas D. Tzanavaras

**Affiliations:** 1 Laboratory of Analytical Chemistry, School of Chemistry, Faculty of Sciences, Aristotle University of Thessaloniki, GR-54124 Thessaloniki, Greece; dimbaltzis@gmail.com (D.B.); gtsogkas@chem.auth.gr (G.Z.T.); 2 Laboratory of Pharmaceutical Analysis, School of Pharmacy, Aristotle University of Thessaloniki, GR-54124 Thessaloniki, Greece; czacharis@pharm.auth.gr

**Keywords:** histidine, 96-well plates, high-throughput assay, human urine, fluorimetry, smartphone detection

## Abstract

A high-throughput fluorimetric assay for histidine was developed, using a 96-well plates platform. The analyte reacts selectively with *o*-phthalaldehyde under mild alkaline conditions to form a stable derivative. Instrumental-free detection was carried out using a smartphone after illumination under UV light (365 nm). The method was proved to be linear up to 100 μM histidine, with an LLOQ (lower limit of quantification) of 10 μM. The assay was only prone to interference from glutathione and histamine that exist in the urine samples at levels that are orders of magnitude lower compared to histidine. Human urine samples were analyzed following minimum treatment and were found to contain histidine in the range of 280 to 1540 μM. The results were in good agreement with an HPLC corroborative method.

## 1. Introduction

The name histidine originates from the Greek word “histion”, which means “tissue”, and was first isolated in 1896 from salmon protamine [1]. Compared to other amino acids, the unique properties of histidine are provided by the imidazolium side chain [2]. Imidazole of histidine is (i) an aromatic motif; (ii) an ionizable group with a p*K*_a_ of around 6.5; (iii) an active ligand capable of coordinating metals; and (iv) a donor and acceptor during the formation of hydrogen bonds [3]. Compared to, e.g., proline and glycine, histidine is reported to be one of the least abundant amino acids in humans, with its concentration being up to 73% in histidine-rich proteins [4]. The determination of histidine in biological material such as urine is a useful tool for the diagnosis of metabolism disorders of the amino acid. For example, “histidinemia” causes elevated levels of the analyte in physiological fluids (normal level: 130–2100 μM in urine) [5], while allergic patients often show higher levels of histidine secretion [6].

A variety of analytical methodologies have been proposed during the last decade for the determination of histidine in complex matrices (food, biological material, cosmetics). An overview of such methods is presented in Table 1 [7,8,9,10,11,12,13,14,15,16,17,18,19]. Ion exchange chromatography coupled to online post-column derivatization offers a powerful tool for the quantification of histidine in complicated matrices with simple sample preparation [7,10,13].

The apparent “disadvantage” of such setups is the rather complicated instrumentation (including reaction coils, pumps, and heaters) and the necessity for familiarity with online reactions under flow conditions. Simple HPLC coupled to UV detection generally suffers from selectivity issues that require additional sample treatment steps before analysis [12], while more sophisticated LC-MS/MS lack widespread availability and have high running and maintenance costs [11]. On the other hand, NMR-based methods are restricted by both the instrumentation and the poor sensitivity at the milli-molar level [9].

Portability and in situ analyses are the most up-to-date demands of modern analytical chemistry. Considering the recent progress in miniaturized sample preparation [20,21] in most of the cases, detection can be considered as the “bottleneck” for the evolution of such portable analytical platforms. Smartphone-based imaging devices (SIDs) are considered as platforms that take advantage of and apply the imaging capabilities of smartphones to applications other than conventional photography [22]. The capabilities of modern smartphones in chemical analysis are not only restricted to their built-in hardware that enables sensing, but scientists have also taken advantage of the computational and rapid sharing potentials of the results [23]. The interested reader can “dig in” to numerous research and especially review articles that discuss the numerous analytical applications of smartphones and related devices in areas such as microfluidics [24,25], food safety [26], instrumentation/software [27], biosensors [28], and diagnostics [29,30,31] just to name a few.

Although paper-based analytical devices (PADs) offer interesting features in terms of portability and smartphone-based detection [8,14,17], their performance and reproducibility are governed by the structural characteristics of the papers, they are not commercially available and, in many cases, require several modification steps before detection. The scope of this research was to develop, evaluate, and apply a high-throughput, 96-well plates-based assay for the selective quantification of histidine in human urine samples. The method takes advantage of the selective and fast reaction of the amino acid with *o*-phthalaldehyde in a basic medium to form a stable fluorescence derivative. Instrumental-free detection was carried out through image capturing using a smartphone after illumination of the plate by a UV lamp at 365 nm.

## 2. Results and Discussion

### 2.1. Preliminary Experiments

OPA reacts with primary aliphatic amines in the presence of nucleophilic compounds (sulfite, mercaptoethanol, N-acetylcysteine) through a non-specific mechanism to form highly fluorescent derivatives. The well-documented advantages of OPA, the commercial availability, and the reasonable cost have established the latter as one of the most widely used derivatizing reagents. A handful of compounds such as histidine, hydrazine, glutathione, and histamine react with OPA through a different, unique mechanism, in the absence of nucleophilic additives in acidic or basic medium to also form fluorescent derivatives. When developing OPA-based methods for these analytes, the advantage of selectivity versus amino acids and biogenic amines is of key importance.

**Figure 1:**
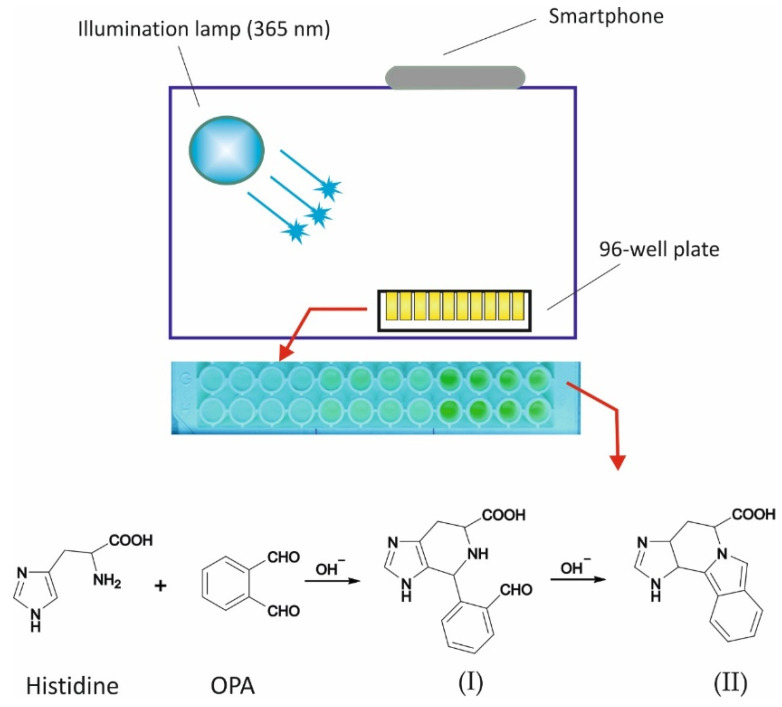
Graphical depiction of the experimental setup for the assay of histidine (including the derivatization reaction [32]). I—non-fluorescent product; II—Fluorescent product.

A representative off-line FL spectra from the reaction between OPA and histidine is shown in Figure S1 (Supplementary Materials). As can be seen in the spectra, the optimum excitation wavelength is λ_ex_ = 360 nm, which is perfectly suitable with the wavelength of the illumination UV lamp. Figure S2 depicts a preliminary image of the plate at 0, 50, and 100 μM histidine (5 mM OPA, pH = 9.0, reaction time 30 min). A graphical depiction of the configuration of the experimental setup (lamp, plate, and smartphone), including the derivatization reaction, is shown in Figure 1.

### 2.2. Development of the Assay for the Determination of Histidine

Chemical variables that were investigated and optimized included the amount concentration of the derivatizing reagent (OPA) in the range of 2 to 20 mM, the effect of the reaction pH in the range of 8 to 10 (100 mM phosphate buffer), and the practical reaction time in the range of 5–30 min. Due to the versatility and the high-throughput potentials of the assay, all experiments were carried out at 5 levels of histidine, namely 10, 25, 50, 75 and 100 μM. In this way, it is possible to study the effect of the variables not only on the sensitivity of the assay but on the linearity as well. The slopes of the respective calibration curves and the linearity were therefore used as optimization criteria for each set of experiments. The illumination and image capture conditions were kept constant in all cases. The volumes of the reagents and samples were also kept fixed at 50/50/150 μL for OPA, buffer, and sample, respectively, corresponding to a total volume of 250 μL per well.

**Figure 2:**
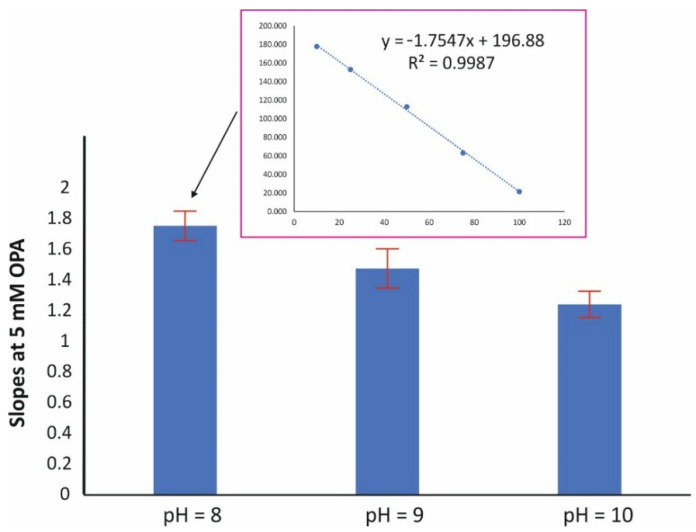
Effect of the pH on the sensitivity of the assay (mean values of slopes ± S.D., *n* = 3); [OPA] = 5 mM, reaction time = 15 min.

**Figure 3:**
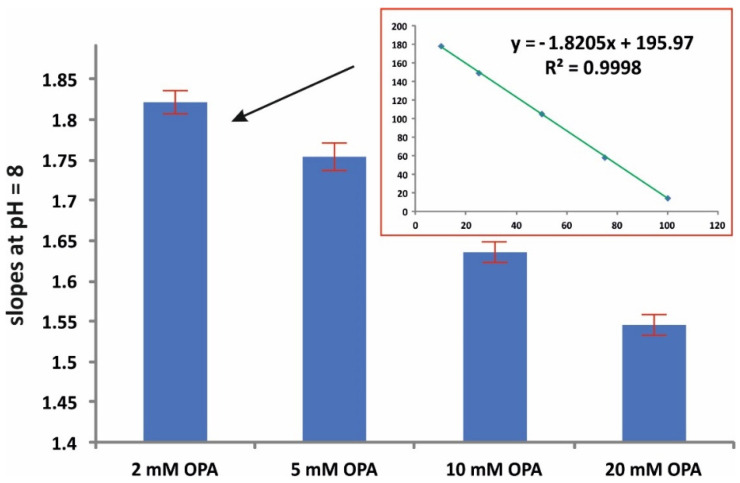
Effect of the amount concentration of OPA on the sensitivity of the assay (mean values of slopes ± S.D., *n* = 3); pH = 8, 15 min reaction time.

Figure 2 depicts the effect of the pH on the assay reaction (at 5 mmol L^−1^ OPA and 15 min reaction time). The pH value of 8 resulted in a higher sensitivity in terms of slope, while good linearity was obtained at all pH values (r^2^ > 0.998). The effect of the amount concentration of the derivatizing reagent was evaluated in the range of 2–20 mM OPA. The pH was set at 8 and the reaction time at 15 min. As can be seen in Figure 3, higher amounts of OPA had a ca 15% negative effect (quenching) on the reaction. The amount concentration of 2 mM was therefore selected for subsequent studies.

Generally, OPA offers rapid reactions with primary amines that are typically completed within a few minutes. On the other hand, the majority of the OPA derivatives suffer from moderate stability. The reaction time was confirmed to have a negligible effect on the performance of the assay for practical values > 10 min, while no stability issues were recorded up to 60 min. All images were therefore captured between 10 and 20 min.

Table 2 includes a synopsis of the examined variables, the investigated range, and the finally selected values.

### 2.3. Validation of the Fluorimetric Assay

The developed assay was validated for linearity, limits of detection (LOD), and lower limit of quantification (LLOQ), within and between-days precision, selectivity, matrix effect, and accuracy.

Linearity was obeyed in the range of 10 (LLOQ)–100 μM L^−1^ (*n* = 6). The regression equation was

FI = −1.75758(± 0.0212) × [histidine] + 196.8(± 1.8)

where FI is the fluorescence intensity as measured by the smartphone and [histidine] is the amount concentration of the analyte in μM. The regression coefficient was *r*^2^ > 0.998 and the percent residuals ranged between −8.2 and +6.7%. The LLOQ was set as the lowest level of the calibration curve (10 μmol L^−1^ histidine), while the LOD was estimated to be 3.4 μM, using the standard deviation of the intercept criterion [33].

The within-day precision was validated by preparing aqueous calibration curves using both the same and different 96-well plates (*n* = 8). As can be seen in Table 3, the relative standard deviation of the slopes was <7%. The between-days precision was validated similarly, by preparing two aqueous calibration curves per day, for four non-consecutive working days. The experimental results are also shown in Table 3, verifying an acceptable RSD value of the slopes of better than 9%.

**Figure 4:**
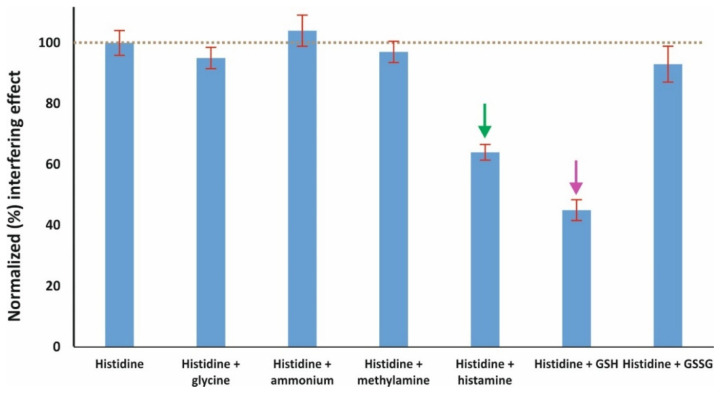
Selectivity of the developed assay; the amount concentration of histidine is 50 μM and of each interferent 100 μM (mean values ± S.D., *n* = 3); the arrows point out the two most important interferences.

Selectivity and matrix effect are—without doubt—the most critical parameters when validating a non-separation method intended to be applied to biological material. In the case of this assay, increased selectivity could be expected due to the unique mechanism of the reaction between histidine and *o*-phthalaldehyde, in the absence of nucleophilic compounds. This mechanism excludes most amino acids, ammonium, and biogenic amines from the formation of fluorogenic derivatives with OPA [18]. All examined potential interferences were examined at 100 μM level + 50 μM histidine and each experiment was performed in triplicate. The following conclusions can be derived from the graphical results shown in Figure 4:

(i) Ammonium and glycine do not seem to have any effect; this could be more or less expected due to the absence of an additional nucleophilic reagent [34].
(ii) The same was observed for the biogenic amine methylamine due to the same reason as mentioned above.
(iii) On the other hand, the biogenic amine histamine that is similarly structured to histidine has a moderate positive interference at 100 μM level. Histamine is also known to react with OPA through the same mechanism, but the derivative is unstable under alkaline conditions (post-reaction acidification is required for stabilization) [32].
(iv) The same moderate effect was observed for the peptide glutathione as well; although glutathione reacts with OPA in the absence of nucleophilic compounds through both the amine and the thiolic group, the optimal pH is >10. On the other hand, no interference was observed from glutathione disulfide, which is known to react with OPA at highly alkaline medium (pH > 12) [35].
(v) Considering the extremely lower levels of glutathione and histamine in urine compared to histidine [36,37], the proposed assay can be considered as selective for the specific application.
(vi) The selectivity results were further evaluated statistically using the *t*-test. As expected, only histamine and glutathione produced statistically different results (*p* < 0.001), while the *p*-values for the rest of the selected interferences ranged between 0.2071 and 0.5543.

The potential matrix effect was validated using both histidine-free artificial urine and pooled human urine. The former was prepared as described in the experimental section and was spiked with known amounts of the analyte after proper dilution as shown in Table 4. Pooled human urine matrix was prepared by mixing equal volumes of six individual samples (*n* = 6). The pooled matrix was diluted 1:25 (the same dilution is applied to the analysis of real samples) and was spiked with known amounts of histidine in the range of 10–50 μM (*n* = 4). As can be seen from the experimental results of Table 4, both artificial and human urine matrices contribute acceptable matrix effects of <10% at dilution factors >25-fold. The aqueous calibration curve was therefore utilized for quantitative analysis in all subsequent experiments.

The accuracy of the assay was validated using both pooled and individual human urine samples. All samples were diluted 25-fold (see experimental section) and spiked with histidine at the 25 and 50 μM levels. The results that are tabulated in Table 5 confirmed the acceptable accuracy of this high-throughput assay, with the percent recovery being in the range of 78–109%.

Finally, the robustness of the proposed method was evaluated at 50 μM histidine by small deliberate variations of critical chemical and geometrical parameters. The experimental conditions and findings are summarized in Table 6. The method proved to be adequately robust with the percent recoveries being in the range of ±12% in all cases (*n* = 3).

### 2.4. Assay of Histidine in Real Urine Samples

**Figure 5:**
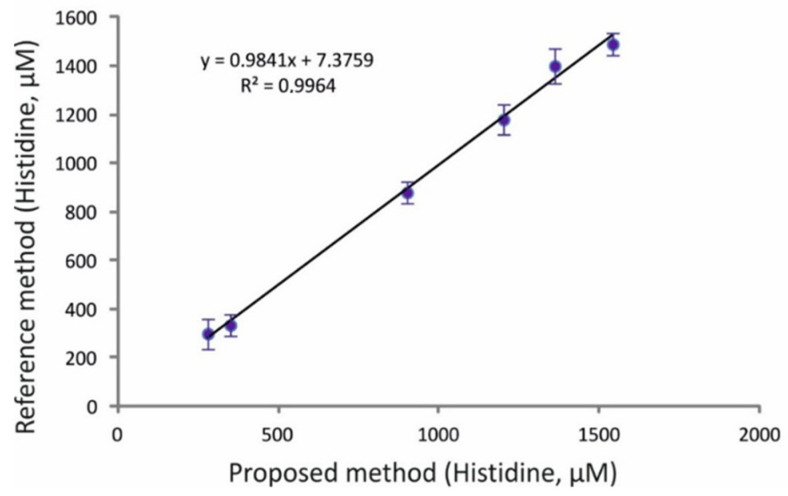
Graphical comparison of the results from the analysis of histidine in real urine samples by the proposed and reference HPLC-PCD methods (mean values ± S.D., *n* = 3; for experimental conditions and details, see text).

The evaluation of the applicability of the proposed high-throughput assay in real-world analyses was based on the determination of histidine in six human urine samples. The collection, preservation, and processing of the samples is described in detail in the experimental section below. As can be seen in the results of Table 7, the concentration of the analyte ranged between 280 and 1540 μM. Table 6 also contains the results from the analysis of the same samples by a corroborative HPLC method. The graphical comparison presented in Figure 5 indicates good correlation between the proposed and reference methods, while representative urine chromatograms from the HPLC method can be seen in Figure S2 (Supplementary Materials). Additionally, the levels of histidine in the urine samples are in good correlation to the reference values for adults (>18 years, *n =* 800) that were reported in the literature [38].

Another series of experiments was carried out to evaluate the stability of the human urine samples at 0, 48, and 72 h; three temperatures were examined, namely 25 °C (room temperature), 4 °C (refrigeration), and −20 °C (freezer). The experimental percent recoveries confirmed the stability of the samples under the selected conditions, ranging between 91 and 114%.

## 3. Materials and Methods

### 3.1. Instrumentation

A VL-115-BL lamb (Vilber-Lourmat) equipped with a 15 W lamp (365 nm) was employed as an excitation source and was placed at an angle of 45° against the well plate. An iPhone 12 pro-max (Apple, Athens, Greece) placed opposite the plate was used for fluorescent image capturing (Figure 1). The 96-well plates for fluorescence were purchased from BrandTech (Thessaloniki, Greece). An RF-5301PC batch spectrofluorophotometer (Shimadzu, Tokyo, Japan) was employed for spectra collection.

### 3.2. Reagents and Solutions

All reagents of this work were at least of analytical grade; histidine (99%, Sigma, Athens, Greece), *o*-phthalaldehyde (OPA, Fluka, Thessaloniki, Greece), HCl (Sigma, Athens, Greece), NaOH (Merck, Thessaloniki, Greece), and KH_2_PO_4_ (Merck, Thessaloniki, Greece). Doubly de-ionized water was obtained from a Milli-Q purification configuration (Millipore, Thessaloniki, Greece).

The analyte stock solution (*c* = 1000 μM) was prepared in water and was stable for at least three days if kept refrigerated at 4 °C. Working standards were freshly prepared by serial dilutions of the stock. The OPA solution (*c* = 2 mM) was prepared in two steps: (i) dissolution of the accurately weighed solid in 0.5 mL methanol, and (ii) subsequent addition of 9.5 mL water [39]. The reagent was stable for 3–4 days at 4 °C and protected from the light. Phosphate buffer (100 mM) was also prepared daily and regulated to the desired pH value (pH = 8.0) by drop-wise addition of NaOH (1 M).

A total of 200 mL of artificial urine was prepared for validation purposes based on the recipe of Brooks and Keevil [40]: lactic acid (1.1 mM), citric acid (2.0 mM), sodium bicarbonate (25 mM), urea (170 mM), calcium chloride (2.5 mM), sodium chloride (90 mM), magnesium sulfate (2.0 mM), sodium sulfate (10 mM), potassium dihydrogen phosphate (7.0 mM), di-potassium hydrogen phosphate (7.0 mM), and ammonium chloride (25 mM). The pH of the solution was adjusted to 6.0 by the addition of 1.0 M HCl.

Selectivity studies were performed using representative similarly structured compounds such as ammonium, glycine (amino acid), methylamine and histamine (biogenic amines), and glutathione and glutathione disulfide (peptides). These reagents were also of analytical grade, were supplied by Sigma, and their solutions were prepared in Milli-Q water.

### 3.3. Assay Procedure

In total, 50 μL of OPA (2 mM), 50 μL buffer (100 mM phosphate, pH = 8.0), and 150 μL of standards (10–100 μM) or urine samples (diluted 1:25) were transferred in each well of the plate. Following orbital mixing for 15 min, the plate was subjected to illumination (365 nm) and the fluorescent image was captured via the smartphone, placed opposite to the plate at a set distance of 20 cm. The photographs were saved as files in JPEG format (300 dpi) and were processed via the Image J program in RGB mode (Image → type → RGB stack → blue).

### 3.4. Preparation of Urine Samples

Urine samples were donated from male and female volunteers (no ethical approval was required) who were informed in detail about the scope of the present research. If not processed immediately, urine samples were stored in air-tight containers at −20 °C [41].

Simple sample processing included protein precipitation with the addition of ice-cold acetonitrile (1 + 1), centrifugation (4000 rpm, 10 min), and dilution with water.

### 3.5. Analysis by an HPLC Method

The corroborative analysis of the real urine samples was carried out by cation exchange HPLC followed by online post-column derivatization (PCD) as described by Stampina et al. [15]. The HPLC-PCD consisted of the following parts: an RF-551 spectrofluorimetric detector operated at the high sensitivity mode (λ_ex_/λ_em_ = 360/440 nm) (Shimadzu, Tokyo, Japan); an AS3000 autosampler (Thermo Scientific, Thessaloniki, Greece); an EliteTM vacuum degasser (Alltech, Athens, Greece); an LC-9A binary pump (Shimadzu, Tokyo, Japan). The PCD reagents were propelled using a MinipulsTM 3 peristaltic pump (Gilson, Athens, Greece). Column ovens (Jones Chromatography, Athens, Greece and HiChrom Limited, Athens, Greece respectively) were used to thermostate the cation exchange column and the reaction coil (0.5 mm i. d. PTFE). Histidine was separated from the matrix through a MetroSep C4 column (150 × 4.0 mm i. d., 5 μm) (Metrohm, Athens, Greece) using 5 mM HNO_3_ at a flow rate of 1.0 mL min^−1^ and a column temperature of 60 °C. The PCD conditions were as follows: OPA (10 mM); phosphate buffer (50 mM pH = 9); 200 cm long reaction coil thermostating at 60 °C, and 0.5 mL min^−1^ for the total flow rate of the reagents. The Clarity^®^ software (version 8.2.1.84, DataApex, Prague, Czech Republic) was utilized for data processing.

## 4. Conclusions

The developed fluorimetric assay for the quantification of histidine offers some interesting features that can be summarized as follows: it utilizes readily available reagents and readily available smartphone-based detection, offering a high-throughput platform by taking into advantage the potentials of 96-well plates. Based on the unique mechanism of the derivatization reaction, the method is highly selective for urine analysis, avoiding interferences from co-existing amino acids (interfering histamine and glutathione do not exist in comparable amounts in the real samples). The sensitivity of the proposed assay is adequate for the direct determination of endogenous histidine in human urine since the absence of matrix effects enables the quantification of histidine using an aqueous calibration curve following moderate dilution of the samples (12.5–25 fold).

## Figures and Tables

**Table 1:** Overview of recent methods for the determination of histidine in real samples.

Method Principle	Detection	Range	Sample/Matrix	Ref
Cation exchange chromatography coupled to post-column derivatization	FL	1.5–30 mg kg^−1^	Hair care products	[7]
Paper-based device	FL	5–40 μM	Urine	[8]
NMR	NMR	2.5–40 mM	Vaccines	[9]
Cation exchange chromatography coupled to post-column derivatization	FL	0.5–5 μM	Human saliva	[10]
HPLC-MS/MS	MS	—	Biological	[11]
HPLC-DAD	UV	0.64–64 μM	Saliva	[12]
Cation exchange chromatography coupled to post-column derivatization	FL	0.5–5 μM	Fisheries	[13]
Paper-based sensor	Vis	1–90 μM	Urine	[14]
Cation exchange chromatography coupled to post-column derivatization	FL	0.5–25 μM	Urine and serum	[15]
Colorimetric sensor	Vis	0.5–3.5 μM	Vegetables	[16]
Paper-based sensor	Vis	1–100 μM	—	[17]
Zone fluidics	FL	0.15–2 μM	Urine	[18]
Sensor	FL	0.05–2.5 μM	Serum	[19]

FL = fluorimetry; NMR = nuclear magnetic resonance; UV = ultraviolet; Vis = visible; GCE = glassy carbon electrode; MS = mass spectrometry.

**Table 2:** Overview of the operating conditions for the assay of histidine.

Parameter	Studied Range	Selected Value
*c*(OPA)/mM	2–10	2
pH (100 mM phosphate)	8–10	8
Reaction time (min)	5–30	10
*V*(OPA)/μL	—	50
*V*(buffer)/μL	—	50
*V*(sample)/μL	—	150

**Table 3:** Within-day and between-days precision.

	Slopes of Aqueous Calibration Curves	
	Within-Day	Between-Days
1	1.8205	1.8805
2	1.7032	1.7038
3	1.9625	1.951
4	1.7192	1.9802
5	1.8835	1.6252
6	1.6136	1.6018
7	1.6326	1.9706
8	1.7256	1.7056
RSD	6.9%	8.9%

**Table 4:** Matrix effect using artificial and real urine.

	Dilution	Slope	Matrix Effect (%)
Aqueous curve	—	−1.75758	—
Artificial urine	1:25	−1.89818	+8
Artificial urine	1:50	−1.66970	−5
Real urine	1:12.5	−2.05637	+17
Real urine	1:25	−1.93334	+10
Real urine	1:50	−1.65212	−6

**Table 5:** Accuracy of the developed assay.

Urine Sample	Added (μM)	Recovery (%)
Pooled–1	25	84
	50	92
Pooled–2	25	78
	50	88
Individual–1	25	82
	50	109
Individual–2	25	106
	50	108

**Table 6:** Robustness of the proposed assay for histidine (50 μΜ).

Parameter	Varied Range	Percent Recoveries *
c(OPA)/mM	1.8–2.2	94–98
pH (100 mM phosphate)	7.8–8.2	98–107
Reaction time (min)	9–11	92–109
V(OPA)/μL	48–52	93–108
V(buffer)/μL	48–52	90–98
V(sample)/μL	145–155	88–110
Smartphone distance from the plate	19–21	92–95

* Based on the intensity at the optimal values.

**Table 7:** Analysis of human urine samples.

	Histidine (μM) ± S.D. (*n* = 3)	
Sample ^a^	Proposed Method	HPLC-PCD [15]
Urine–1	350 ± 25	335 ± 15
Urine–2	280 ± 23	300 ± 15
Urine–3	1200 ± 60	1180 ± 45
Urine–4	900 ± 45	875 ± 30
Urine–5	1540 ± 85	1490 ± 55
Urine–6	1360 ± 70	1410 ± 50

^a^ Samples were diluted 1:25 and 1:200 for the proposed and reference methods, respectively.

## Data Availability

The data presented in this study are available on request from the corresponding author.

## Supplementary Materials

The following are available online at https://www.mdpi.com/article/10.3390/molecules28176205/s1, Figure S1: Fluorescence spectra of the histidine-OPA derivative. Figure S2: Image from preliminary experiments (0, 50, and 100 μmol L^−1^ histidine, reaction time 30 min, *n* = 8). Figure S3: Representative chromatograms from the application of the corroborative HPLC method (for experimental details, please see Section 2.4 and Section 3.5).

## Sample Availability

Not available.
