# Supplementary material for: Smartphone-Based High-Throughput Fluorimetric Assay for Histidine Quantification in Human Urine Using 96-Well Plates
Source: Molecules. 2023 Aug 23;28(17):6205. doi: 10.3390/molecules28176205 (PMC10488697; doi:10.3390/molecules28176205)
Supplement: Supplementary file 1 [file molecules-28-06205-s001.zip › molecules-2575806-supplementary.pdf]

molecules

Supplementary Material

Smartphone-Based High-Throughput Fluorimetric Assay for Histidine Quantification in Human Urine Using 96-Well Plates

Dimitrios Baltzis [1], George Z. Tsogas [1], Constantinos K. Zacharis [2] and Paraskevas D. Tzanavaras [1,*]

[1] Laboratory of Analytical Chemistry, School of Chemistry, Faculty of Sciences, Aristotle University of Thessaloniki, GR-54124 Thessaloniki, Greece

[2] Laboratory of Pharmaceutical Analysis, School of Pharmacy, Aristotle University of Thessaloniki, GR-54124 Thessaloniki, Greece

* Correspondence: ptzanava@chem.auth.gr; Tel.: +30-23-1099-7721; Fax: +30-23-1099-7719

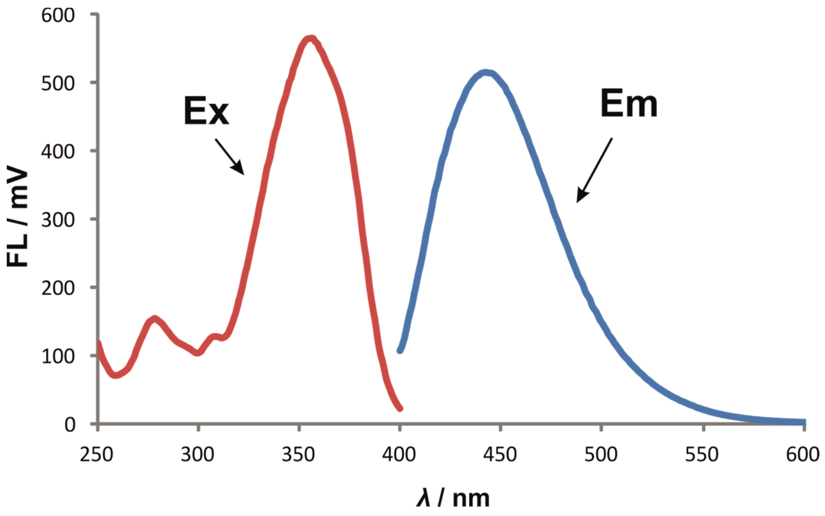

Figure S1. Fluorescence spectra of the histidine-OPA derivative.

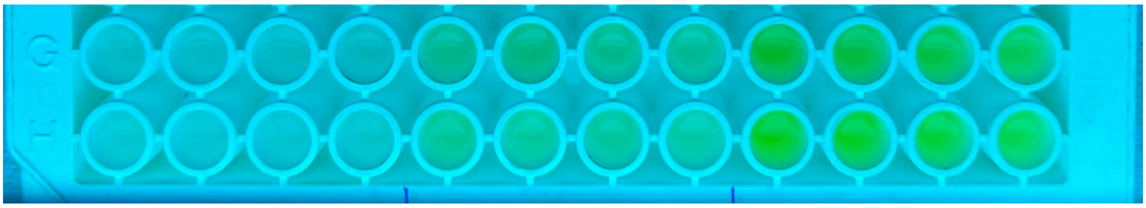

Figure S2. Image from preliminary experiments (0, 50 and100 μmol L^{-1} histidine, reaction time 30 min, $n = 8$).

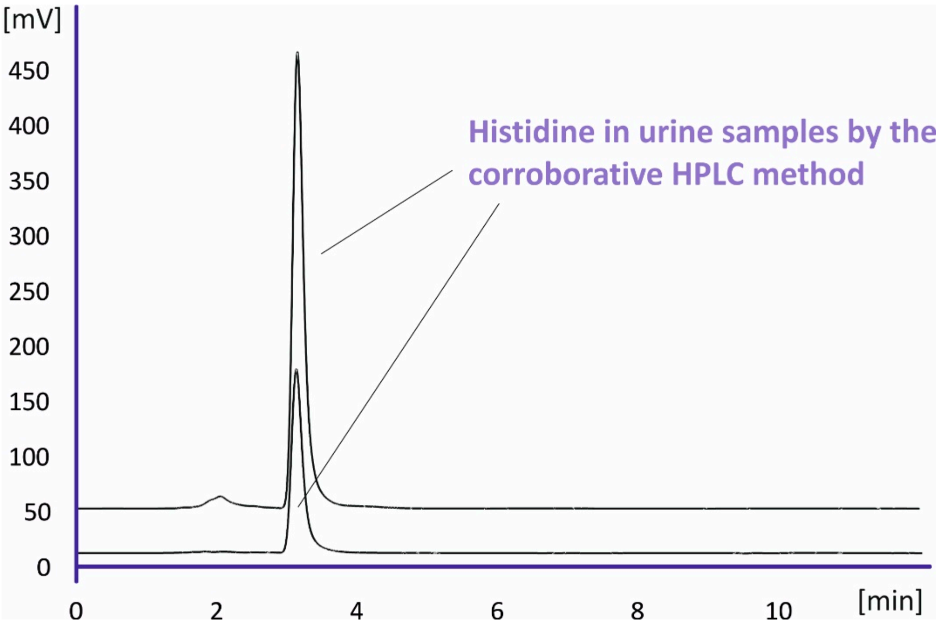

Figure S3. Representative chromatograms from the application of the corroborative HPLC method (for experimental details please see sections 2.4 and 3.5).
